# Clear-Cell Renal Cell Carcinoma Molecular Subtypes Differ by African and European Genetic Similarity

**DOI:** 10.1158/2767-9764.CRC-24-0624

**Published:** 2025-05-01

**Authors:** Roy Elias, Thomas Nirschl, Michael Rezaee, Anirudh Yerrapragada, Shirley Wang, Joseph Cheaib, Ridwan Alam, Sunil Patel, Yuezhou Jing, Mohamad Allaf, David McKean, Alison P. Klein, Elana J. Fertig, Ezra Baraban, Yasser Ged, Srinivasan Yegnasubramanian, Nirmish Singla

**Affiliations:** 1 Sidney Kimmel Comprehensive Cancer Center, Johns Hopkins University School of Medicine, Baltimore, Maryland.; 2 Brady Urologic Institute, Johns Hopkins University School of Medicine, Baltimore, Maryland.; 3 Department of Epidemiology, Johns Hopkins University School of Public Health, Baltimore, Maryland.; 4 Department of Medicine, Johns Hopkins University School of Medicine, Baltimore, Maryland.; 5 Department of Pathology, Johns Hopkins University School of Medicine, Baltimore, Maryland.; 6 Convergence Institute, Johns Hopkins University School of Medicine, Baltimore, Maryland.; 7 Department of Biomedical Engineering, Johns Hopkins University School of Medicine, Baltimore, Maryland.; 8 Department of Applied Mathematics and Statistics, Johns Hopkins University Whiting School of Engineering, Baltimore, Maryland.; 9 inHealth Precision Medicine program, Johns Hopkins Medicine, Baltimore, Maryland.

## Abstract

Self-reported Black (B) individuals remain underrepresented in molecular studies of clear-cell renal cell carcinoma (ccRCC) relative to White (W) individuals. We performed whole-exome and transcriptome sequencing on paired tumor and normal samples from 59 matched B and W patients undergoing nephrectomy for localized ccRCC, comparing molecular differences by estimated genetic similarity to African (AFR) and European (EUR) 1000 Genomes groups. We validated our findings with a propensity-matched subset of The Cancer Genome Atlas, yielding a final cohort of 254 patients (79 AFR and 175 EUR) with similar baseline clinical variables. Significant differences emerged in *VHL* mutation frequency (AFR: 23.4%, EUR: 57.5%; FDR = 0.0029) and chromosome 3p deletions (AFR: 59.2%, EUR: 82.6%; FDR = 0.086). Transcriptomic analyses identified 34 genes associated with genetic similarity, and gene set enrichment revealed inflammatory (IFN-γ/IFN-α, allograft rejection), proliferative (E2F targets, G_2_–M checkpoint), and metabolic (bile acid, fatty acid, glycolysis, MTORC1, peroxisome) pathway enrichment in EUR. We also observed differences in ccRCC molecular subtype distribution, with “Proliferative” and “Angio/Stromal” subtypes being more common in AFR (*P* = 0.018). Importantly, differential subtype membership explained most group-level differences. These results link EUR and AFR genetic similarity to distinct ccRCC molecular subtypes, underscoring the importance of molecular classifiers in disease stratification and the need to include diverse populations in molecular studies to improve our understanding and treatment of ccRCC.

**Significance:**

Our study shows that AFR genetic similarity correlates with distinct ccRCC molecular subtypes. Further research is needed to disentangle environmental and genetic influences. Identifying these differences underscores the critical importance of including racially and ethnically diverse populations in cancer research to ensure more equitable and sustainable outcomes worldwide for all patients.

## Introduction

Renal cell carcinoma (RCC) is among the most frequently diagnosed cancers, with more than 80,000 new cases and nearly 15,000 deaths estimated in the United States in 2024 (1). The incidence of RCC varies by self-reported race, with Black (B) patients demonstrating a higher age-adjusted incidence than White (W) patients (2). The World Health Organization has defined >20 histologically and molecularly distinct subtypes of RCC, with clear-cell RCC (ccRCC) being the most frequent, accounting for approximately 75% of new diagnoses (3). Among patients with RCC, the distribution of histologic subtypes has been shown to vary by race, with B race associated with a decreased frequency of ccRCC but increased frequencies of papillary and MiT family translocation RCC (4–6). Among patients with ccRCC, B race was associated with a decreased frequency of *VHL* mutations, the most frequent alteration in this subtype (7, 8). Collectively, these findings suggest that race may influence the molecular pathogenesis of RCC.

Race is a social construct and is associated with various factors that can conceivably influence ccRCC biology, as outlined in detail in a recent report by the National Academy of Sciences. Genetic ancestry attempts to describe components of an individual’s genetic origin, but in practice, patients are categorized on the basis of genetic similarity and typically labeled by geographical, ethnic, or other nongenetic labels (9). For this reason, the preferred descriptor is “genetic similarity,” which we adopt in this article except when referencing prior studies that use different terminology. The influence of germline genetics on tumor biology has been investigated in The Cancer Genome Atlas (TCGA), in which a decreased frequency of *VHL* and *PBRM1* mutations was identified among patients with elevated African (AFR) ancestry, relative to European (EUR) ancestry (10). Transcriptomic analyses in the same cohort identified decreased estimated immune infiltration and lower hypoxia signaling in AFR versus EUR ancestry groups (10, 11). Despite these observations, efforts to understand the influence of genetics on tumor biology in RCC remain limited by the relative underrepresentation of minority patients in large-scale sequencing studies and clinical trials (12).

In this study, we sought to systematically interrogate the somatic alterations and transcriptomic differences by genetic similarity to AFR and EUR geographic groups as defined by the 1000 Genomes Project across individuals with ccRCC (13). We report an integrative analysis of whole-exome sequencing (WES) and RNA sequencing (RNA-seq) using a case–case-matched cohort of B and W patients balanced by clinicopathologic confounders. We validated our findings in our cohort with a propensity-matched subset of the TCGA, correcting for previous imbalances in clinical variables. Finally, we examine AFR- and EUR-associated molecular differences in the context of clinically relevant molecular classifiers.

## Materials and Methods

### Patients

Institutional Review Board approval was obtained (protocols IRB00204473 and NA_00087094). Patients with localized, pathologically confirmed ccRCC who underwent partial or radical nephrectomy in our institutional experience were queried. Race was self-reported using a multiple-choice question as part of routine medical intake. We selected 30 patients who self-reported as B, and they were case-matched 1:1 with self-reported W individuals, balancing for stage, sex, nuclear grade, and age. Sex was determined using self-reported data from the medical records and included as a variate in all models. Clinical variables were extracted through chart review of the electronic medical records.

### Tissue processing

Tumor and normal adjacent tissue regions from nephrectomy specimens were identified on hematoxylin and eosin–stained slides by a genitourinary board–certified pathologist (E. Baraban). These areas were macrodissected, and DNA/RNA was extracted from formalin-fixed, paraffin-embedded tissue sections using a DNA/RNA FFPE tissue kit (AllPrep DNA/RNA, Qiagen).

### WES

Libraries for WES were constructed using the Agilent SureSelect version 5, beginning with a minimum of 200 ng of genomic DNA, and the Illumina SureSelectXT Target Enrichment System for Illumina Paired-End Multiplexed Sequencing Library version D1 for Illumina Paired-End Multiplexed Sequencing, according to the manufacturer’s instructions. Paired-end sequencing was performed using the NovaSeq 6000 S4 flow cell with 2 × 150 cycle chemistry with an output of approximately 12 Gb per sample. FASTQ files were generated from the sequencer’s output using Illumina bcl2fastq2 version 2.17.1.14 (RRID:SCR_015058) with default filters to select sequence reads for subsequent analyses. Trim Galore version 0.6.7 (RRID:SCR_011847) was used to trim off the adaptor sequences and low-quality bases from the reads. Sentieon 202010.02 release (BWA-MEM version 0.7.17; RRID:SCR_010910) was used for running the alignments against the genome. Sentieon 202010.02 release (RRID:SCR_025615; Picard tools MarkDuplicates version 2.9.0, GATK IndelRealigner version 3.8.0, GATK BaseRecalibrator version 3.8.0, and GATK PrintReads version 3.8.0) was used to create a recalibrated BAM file. Samtools version 1.10 (RRID:SCR_002105) and GATK DepthOfCoverage version 3.8.0 (RRID:SCR_001876) were used to determine coverage at different levels of partitioning and aggregation. Sentieon 202010.02 release (GATK Mutect2 version 3.8.0) was used to call somatic variants between the tumor–normal pairs. Sentieon 202010.02 release (GATK HaplotypeCaller version 3.8.0) was used to call germline variants in each sample. CNVkit version 0.9.4 (RRID:SCR_021917) was used for copy-number variant detection. Passed somatic and germline variants were converted into Mutation Annotation Format files using vcf2maf version 1.6.19. For samples without a matched normal (*n* = 10), putative somatic mutations were compared with reference genomes from the ExAC (RRID:SCR_004068) and gnomAD (RRID:SCR_014964) databases and included only if they were novel or detected at a frequency of <1%. For downstream analyses, only nonsynonymous mutations in coding regions were included. For tumor–normal pairs, the matched normal was used, and for tumor-only samples (*n* = 9), a flat reference was used. A cutoff of −0.3 log_2_ fold change across segments was used to call copy-number loss, and a cutoff of 0.3 log_2_ fold change across segments was used to call copy-number gain.

### Genetic similarity estimation

We followed guidance from the National Academies consensus statement “Using Population Descriptors in Genetics and Genomics Research, 2023” about population descriptors in this study. This statement specifically provides flowcharts recommending the appropriate terminology depending on the type of study (Page 199). When referencing other studies, we utilized the population descriptors defined in those studies (i.e., race, ancestry). Germline variants from the 1000 Genomes Project (RRID:SCR_006828; ref. 14) were downloaded and pooled with germline variants from the WES cohort using bcftools version 1.18 (RRID:SCR_005227; https://samtools.github.io/bcftools/bcftools.html). Binary .bed files of the pooled cohort were generated using PLINK version 2.0 (RRID:SCR_001757; https://www.cog-genomics.org/plink/2.0/). We pruned variants for linkage disequilibrium by excluding those with an r^2^ > 0.2 within a sliding window of 50 SNPs, shifting 5 SNPs per step. These files were used as input for ADMIXTURE (RRID:SCR_001263; https://dalexander.github.io/admixture/), which was run with default settings and k = 5 populations, the number of superpopulations described in the 1000 Genomes Project. This produced an estimated percentage of AFR, EUR, South Asian, East Asian, and American genetic similarity per sample. For a patient-level genetic similarity call, we deferred to germline calls performed on adjacent normal specimens unless these were unavailable, in which case we utilized HaplotypeCaller calls on tumor specimens. AFR and EUR groups were defined using hierarchical clustering on genetic similarity percentages.

### RNA-seq

Sequencing libraries were constructed using the Illumina TruSeq RNA Exome kit (Illumina) for 150-bp paired-end reads, and RNA-seq was aligned and sequenced using HiSeqX (150 bp PE). FASTQ files were generated from the sequencer’s output using Illumina bcl2fastq2 version 2.17.1.14 (RRID:SCR_015058) with default filters to select sequence reads for subsequent analyses. Trim Galore version 0.6.7 (RRID:SCR_011847) was used to trim off the adaptor sequences and low-quality bases from the reads. The reads were aligned to the human reference genome (hg38) in a splice-aware fashion using RSEM version 1.3.0 (RRID:SCR_000262) with STAR (RRID:SCR_004463) as the aligner. RSEM was also used for generating gene-level expression estimates.

### TCGA patient cohort

Specimen and patient IDs of ccRCC cases in the TCGA (RRID:SCR_003193) were accessed from the Genomic Data Commons (https://gdc.cancer.gov/; *n* = 537). We filtered for cases with somatic mutations, segmented copy-number calls, RNA-seq data available, and estimated genetic ancestry (*n* = 356; ref. 10). Next, we excluded cases that had been reclassified as non-ccRCC (*n* = 15; ref. 15, 16). From this cohort, there were 49 patients with either predominantly AFR or AFR admixed ancestry. We selected a propensity-matched cohort of patients with primary EUR ancestry using the R software “MatchIt” version 4.5.4 (RRID:SCR_025618; https://github.com/kosukeimai/MatchIt) balanced on age at diagnosis, gender, nuclear grade, and disease stage at a 1:3 AFR/EUR ratio. Somatic mutation calls, segmented copy-number calls, and RNA expression data were downloaded directly from the NCI Genomic Data Commons (RRID:SCR_014514).

### Analysis of RNA-seq data

Differential expression analysis was performed using the DESeq2 version 3.20 (RRID:SCR_015687) R package (17). The model included tissue type (tumor vs. normal) and AFR/EUR group in a concatenated variable, allowing the multiple comparisons seen in Fig. 3 and Supplementary Fig. S3. Gene set enrichment analysis (GSEA) was performed using the fgsea version 1.26.0 (RRID:SCR_020938) R package (bioRxiv 2021.060012) on ranked outputs from differential expression analysis. Hallmark gene sets (version 2022.1) were downloaded from the Molecular Signatures Database (RRID:SCR_016863; https://www.gsea-msigdb.org/gsea/msigdb; ref. 18). For visualization across TCGA and Johns Hopkins University (JHU) cohorts, batch correction of log_2_ transformed transcripts per million (TPM) was performed using the “ComBat” function of the SVA version 3.48.0 (RRID:SCR_002155) R package (19). Sample-level gene set variation analysis (GSVA) was performed using the GSVA version 1.48.3 (RRID:SCR_021058) R package on batch-corrected log_2_ transformed TPM values (20). Cellular deconvolution was performed using xCell (https://comphealth.ucsf.edu/app/xcell; ref. 21) on batch-corrected log_2_ transformed TPM values.

### Predicting IMm151 clusters

We obtained log_2_ TPM data and cluster annotations for 823 samples from the IMmotion151 (IMm151) phase III study (22) from the European Genome-phenome Archive (RRID:SCR_004944; EGAD00001006617 and EGAD00001006618). We trained a random forest model on these data to predict clusters in other datasets as previously described (22). Briefly, we first subsetted the gene matrix to the top 10% of genes by median absolute deviation (*n* = 3,072). This matrix was further subsetted to the intersection of genes in the JHU and TCGA cohorts (*n* = 2,731). Samples classified as “small nucleolar (sno) RNA” (*n* = 28) were excluded because of limited coverage of noncoding RNAs in the TCGA cohort. We split the IMm151 data into training and testing datasets at an 80:20 split, with preservation of cluster frequencies. A random forest model was tuned using the caret version 6.0-94 (RRID:SCR_022524) and ranger version 0.15.1 (RRID:SCR_022521) R packages. Model performance was assessed in the testing dataset, and the optimal parameters (m.try = 50, min.node.size = 10) were applied in the final model. The performance of the model was assessed in the test cohort and then applied to the batch-corrected log_2_ TPM values of the study dataset.

### Statistical consideration

All statistical analyses were performed using R version 4.3.1 running in RStudio version 2023.06.0 (RRID:SCR_000432). Comparisons of proportions were performed using Fisher’s exact test or χ^2^ test. Comparison of medians between groups was performed using the Kruskal–Wallis test. Comparisons of mutation status with AFR percentage were performed using logistic regression analysis. *P* values were adjusted for multiple comparisons using Benjamini–Hochberg corrections. Significance was defined as *P* < 0.05. Figures (bar plots, box plots, and scatter plots) were generated using ggplot2 version 3.4.2 (RRID:SCR_014601). OncoPrints and heatmaps were generated using ComplexHeatmap version 2.16.0 (RRID:SCR_017270; ref. 23).

### Data availability

All source codes utilized to generate the described analysis are deposited on GitHub at the following repository (https://github.com/rmelias2/rcc_gen_sim). Counts and relevant clinical data for the JHU cohort have been submitted to the Gene Expression Omnibus (GSE289907)*.* Processed WES calls are available in the supplementary material. Because of informed consent limitations on patient confidentiality and secondary use of data, restrictions apply to the availability of the raw WES and RNA-seq data. Anonymized data can be made available from the authors under a Data Access Agreement upon reasonable request and with the permission of the Institutional Review Board.

## Results

### Patient cohorts and genetic similarity estimation

We identified a case–case race-matched cohort of 66 patients (*n* = 34 B, *n* = 32 W) with ccRCC who underwent surgical resection at our institution from 2004 to 2020. Cases were matched on sex, stage, and nuclear grade. Available tissue [*n* = 123 samples (57 tumor–normal pairs and 9 tumor-only)] was submitted for WES and RNA-seq. Following quality control (Supplementary Fig. S1A), our institutional cohort (JHU cohort) consisted of 59 patients with either tumor WES (*n* = 42) or tumor RNA-seq (*n* = 58) data available (Table 1). There were no statistically significant differences in average coverage for WES or total mapped reads in RNA-seq between B and W individuals (Supplementary Fig. S1B and S1C). Clinical characteristics were similar between the two groups; however, the estimated glomerular filtration rate (eGFR) and hypertension (HTN) were numerically higher in self-reported B individuals relative to W individuals. Genetic similarity was estimated using ADMIXTURE with the 1000 Genomes Project as a reference cohort (13, 24). Unsupervised clustering of estimated genetic similarity revealed two subgroups corresponding to predominantly EUR (*n* = 28) and AFR (*n* = 30) geographic groups (Supplementary Fig. S1D; Supplementary Table S1).

**Table 1:** Clinicopathologic characteristics of case–case-matched cohort

	Total	B	W	*P* value
	(*n* = 59)	(*n* = 30)	(*n* = 29)	
Age	59.2 (±12.3)	60.6 (±13.1)	57.8 (±11.6)	0.36
Sex				0.44
Female	31 (52.5%)	14 (46.7%)	17 (58.6%)	
Male	28 (47.5%)	16 (53.3%)	12 (41.4%)	
eGFR	69.4 (±27.2)	63.3 (±31.8)	75.6 (±20.2)	0.12
HTN Hx				0.16
No	17 (28.8%)	6 (20.0%)	11 (37.9%)	
Yes	42 (71.2%)	24 (80.0%)	18 (62.1%)	
pT stage				0.6
pT1	37 (62.7%)	20 (66.7%)	17 (58.6%)	
pT3	22 (37.3%)	10 (33.3%)	12 (41.4%)	
Grade				0.49
2	39 (66.1%)	21 (70.0%)	18 (62.1%)	
3	19 (32.2%)	8 (26.7%)	11 (37.9%)	
4	1 (1.7%)	1 (3.3%)	0 (0.0%)	
Estimated genetic similarity				**<0.0001**
AFR	30 (40.7%)	24 (80.0%)	0 (0.0%)	
EUR	28 (47.5%)	0 (0.0%)	28 (96.6%)	
Not available	1 (1.7%)	1 (3.3%)	0 (0.0%)	
RNA-seq available				1
Passed	58 (98.3%)	29 (96.7%)	29 (100.0%)	
Failed	1 (1.7%)	1 (3.3%)	0 (0.0%)	
WES available				1
Matched normal	33 (55.9%)	17 (56.7%)	16 (55.2%)	
Pooled normal	9 (15.3%)	4 (13.3%)	5 (17.2%)	
Failed	17 (28.8%)	9 (30.0%)	8 (27.6%)	

*P* values < 0.05 are in bold.

Abbreviation: pT stage, pathologic T stage.

We also curated a propensity-matched subset of the ccRCC TCGA cohort (TCGA Project ID: KIRC; Supplementary Fig. S2). Recent studies have identified non-ccRCC cases among the KIRC dataset, and these were present in a greater proportion of patients with AFR ancestry (15, 16). After excluding non-ccRCC cases, we identified 49 TCGA-KIRC cases with AFR or AFR-admixed ancestry (defined as patients with majority AFR ancestry but less than 80% in the TCGA; ref. 10) and matched 1:3, controlling for sex, age, tumor stage, and nuclear grade, for a final cohort of 196 patients (referred to as the TCGA cohort). Unsupervised clustering of estimated genetic similarity in the TCGA cohort was consistent with the results of the JHU cohort, revealing two clusters corresponding to EUR (*n* = 147) and AFR (*n* = 49) groups (Supplementary Fig. S2). In both the JHU and TCGA cohorts, AFR genetic similarity was strongly associated with self-reported B race, and EUR genetic similarity was strongly associated with W race (*P* < 0.001).

### AFR/EUR group, but not percentage AFR genetic similarity, is associated with a decreased frequency of *VHL* mutations and chr3p deletions

**Figure 1:**
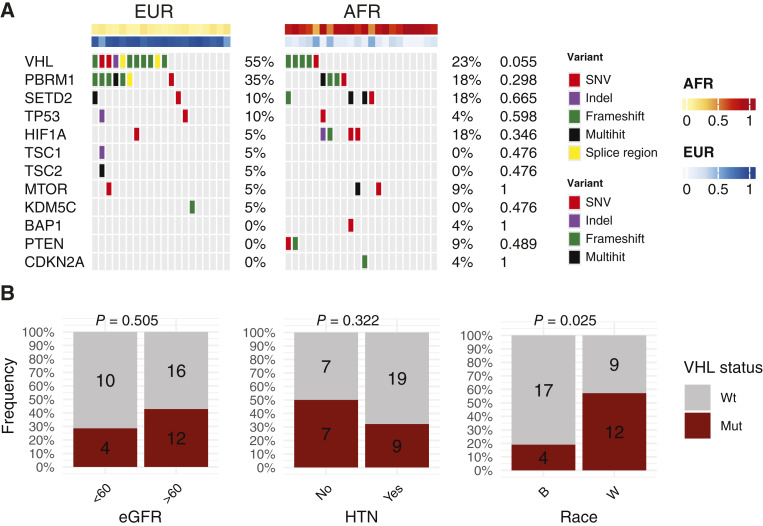
ccRCC driver mutations by AFR and EUR groups, race, and RCC clinical risk factors. **A,** OncoPrint of ccRCC driver mutations split by AFR and EUR groups. **B,** Frequency of *VHL* mutations in patients by HTN history, eGFR group, and self-reported race. Group-wise *P* values were calculated using Fisher’s exact test. Mut, mutant; SNV, single nucleotide variant; Wt, wild-type.

WES was available for 42 patients in the JHU cohort (*n* = 20 EUR and *n* = 22 AFR). Given the limited sample size, we focused our analysis on known driver mutations in ccRCC. We observed an elevated frequency of *VHL* mutations in the EUR group although this did not reach statistical significance (EUR = 55% vs. AFR = 23%, *P* = 0.055; Fig. 1A; Supplementary Table S2). The frequency of other ccRCC driver mutations, including *PBRM1*, *SETD2*, and *BAP1*, was similar between the two groups. Genetic similarity to AFR or EUR groups, as determined by the 1000 Genomes Project, is strongly associated with race (13). We assessed whether differences in renal function and HTN status, two clinical risk factors for ccRCC that are more prevalent among B individuals, could account for the differences observed in VHL frequency between the AFR and EUR cohorts (25). *VHL* mutation rates were similar between eGFR groups (eGFR < 60 = 29% vs. eGFR > 60 = 43%, *P* = 0.51) and HTN history (HTN = 32% and no HTN = 50%; *P* = 0.32) but were significantly associated with race (B = 19% and W = 57.1%; *P* = 0.025; Fig. 1B), suggesting that race, but not differing frequencies of HTN and chronic kidney disease, accounts for the difference in *VHL* mutation frequency observed between AFR and EUR groups.

**Figure 2:**
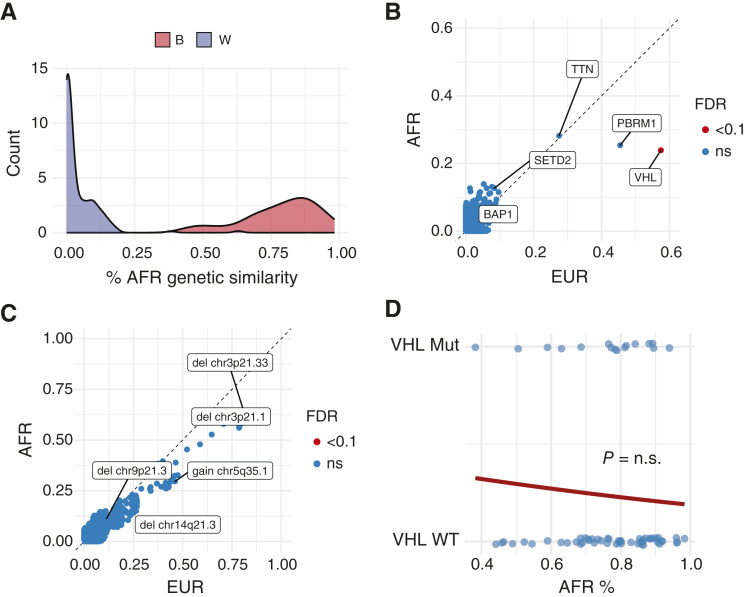
AFR and EUR groups drive somatic alterations in ccRCC independent of AFR genetic similarity percentage. **A,** Density plot of AFR genetic similarity by self-reported race demonstrating a bimodal distribution. Frequency of somatic mutations (**B**) and cytoband gains/losses (**C**). *P* values were determined by Fisher’s exact test and corrected for FDR. **D,** Logistic regression of AFR percentage among B individuals (*n* = 69) vs. *VHL* mutation status. *P* values were calculated by logistic regression and adjusted for multiple hypothesis testing by Benjamini–Hochberg. n.s., not significant.

Next, to increase statistical power, we pooled our institutional (*n* = 58 with available genetic similarity estimation) cohort with the propensity-matched subset of the TCGA ccRCC cohort (*n* = 196) described above and performed a genome-wide comparison of somatic variants and copy-number gains and losses. The pooled analytic cohort consisted of 254 patients, of whom 238 had available tumor WES (EUR = 167 and AFR = 71) and was balanced on sex, age, disease stage, and grade (Table 2; Supplementary Table S3). AFR genetic similarity demonstrated a bimodal distribution by race, consistent with the observation that AFR and EUR levels of genetic similarity are strongly confounded by race (Fig. 2A). Given this distribution, we elected to compare somatic alteration frequency first across AFR and EUR groups, followed by the AFR percentage among patients with self-reported B race. Following FDR correction, the frequency of *VHL* mutations was lower in individuals of AFR genetic similarity (23.4%) compared with those of EUR genetic similarity (57.5% FDR = 0.0029), as were deletions across chr3p (chr3p21.33: AFR = 59.2%, EUR = 82.6%, FDR = 0.086; Fig. 2B and C). *PBRM1* mutations were more common in EUR patients (45.5%) versus AFR patients (25.4%), but this did not meet statistical significance following FDR correction in this propensity-matched and pooled cohort (FDR = 0.55). We next compared AFR percentage with *VHL* mutation frequency among patients with self-reported B race (*n* = 69). We found that AFR percentage was not statistically associated with *VHL* mutation frequency (OR = 0.31, *P* = 0.5) among patients with self-reported B race (Fig. 2D). This suggests that the observed differences in *VHL* mutation frequency are associated with the AFR group, not the percentage of AFR genetic similarity among B individuals, although a small sample size could influence this analysis.

**Table 2:** Pooled analytic cohort

	Total	AFR	EUR	
	(*n* = 254)	(*n* = 79)	(*n* = 175)	*P* value
Age	59.3 (±12.3)	59.7 (±11.6)	59.2 (±12.6)	0.75
Sex				0.79
Female	115 (45.3%)	37 (46.8%)	78 (44.6%)	
Male	139 (54.7%)	42 (53.2%)	97 (55.4%)	
Stage				0.79
Stage I	173 (68.1%)	54 (68.4%)	119 (68.0%)	
Stage II	15 (5.9%)	4 (5.1%)	11 (6.3%)	
Stage III	39 (15.4%)	14 (17.7%)	25 (14.3%)	
Stage IV	25 (9.8%)	6 (7.6%)	19 (10.9%)	
NA	2 (0.8%)	1 (1.3%)	1 (0.6%)	
Grade				0.93
1	13 (5.1%)	4 (5.1%)	9 (5.1%)	
2	126 (49.6%)	41 (51.9%)	85 (48.6%)	
3	101 (39.8%)	29 (36.7%)	72 (41.1%)	
4	9 (3.5%)	3 (3.8%)	6 (3.4%)	
NA	5 (2.0%)	2 (2.5%)	3 (1.7%)	
Cohort				**0.0002**
JHU	58 (22.8%)	30 (38.0%)	28 (16.0%)	
TCGA	196 (77.2%)	49 (62.0%)	147 (84.0%)	
WES available				0.1
Passed	238 (93.7%)	71 (89.9%)	167 (95.4%)	
Failed	16 (6.3%)	8 (10.1%)	8 (4.6%)	
RNA-seq available				0.31
Passed	253 (99.6%)	78 (98.7%)	175 (100.0%)	
Failed	1 (0.4%)	1 (1.3%)	0 (0.0%)	

*P* values < 0.05 are in bold.

### Transcriptomic analyses reveal distinct oncogenic pathways and tumor microenvironment composition associated with AFR and EUR genetic similarity

Next, we assessed the interaction of AFR/EUR genetic similarity with tumor and normal kidney by comparing gene expression across multiple groups: AFR tumor (AFR T, *n* = 30), AFR normal (AFR N, *n* = 25), EUR tumor (EUR T, *n* = 28), and EUR normal (EUR N, *n* = 19). Using an FDR *P* value cutoff of <0.05 and an absolute log fold change >0.5, we identified 3,765 differentially expressed genes (DEG) in AFR T versus AFR N (1,801 upregulated in AFR T and 1,964 upregulated in AFR N) and 5,667 DEGs in EUR T versus EUR N (2,930 upregulated in EUR T and 2,737 upregulated in EUR N; Supplementary Table S4). As expected, there was a strong correlation between the DEGs identified in these two comparisons, indicating that tumor versus normal gene expression differences are largely similar, independent of AFR/EUR grouping. Hallmark GSEA of the AFR T versus AFR N and EUR T versus EUR N DEG lists revealed high concordance in pathway significance and direction between the two comparisons (32 out of 50 hallmark gene sets, *P* < 0.0001). Classical ccRCC pathways—including IFN-γ response, hypoxia, glycolysis, and MTORC1 signaling—were among the upregulated gene sets in tumor versus normal comparisons across both AFR and EUR groups (Supplementary Fig. S3).

**Figure 3:**
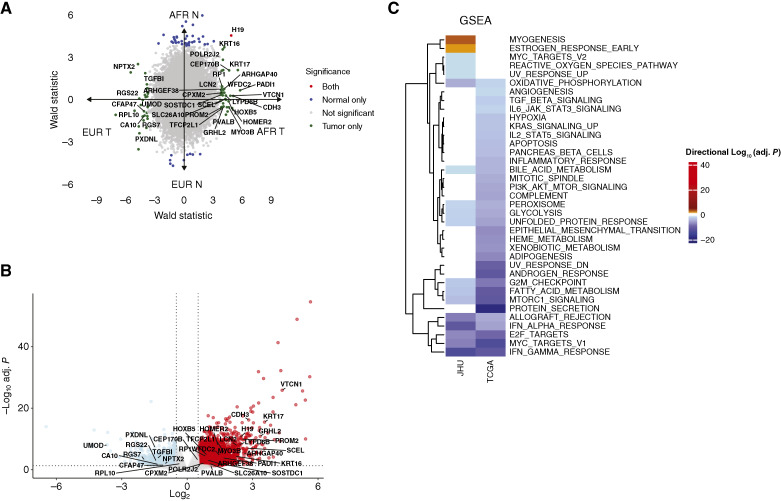
Differential expression analysis and hallmark gene set enrichment of AFR vs. EUR groups. **A,** Wald statistic of DEGs comparing AFR T vs. EUR T (*x*-axis) and AFR N vs. EUR N (*y*-axis). Colored points depict significant genes with an FDR-adjusted *P* < 0.05, with green representing genetic similarity–associated genes in tumor comparisons (*n* = 73), blue representing genetic similarity–associated genes in normal comparisons (*n* = 74), and red representing genes that are significant in both comparisons (*n* = 1). **B,** Volcano plot depicting DEGs in AFR vs. EUR in the TCGA cohort (*n* = 8,739 DEGs, 4,952 AFR-associated, 3,787 EUR-associated). DEGs in **A** and **B** are defined as having an FDR-adjusted *P* value < 0.05 and an absolute log_2_ fold change >0.5. Labeled points are significantly altered in both JHU and TCGA comparisons (*n* = 34). **C,** Heatmap of hallmark gene set enrichment results performed on the DEGs produced in **A** and **B**. Plotted values are directional [−log_10_(adj. *P*)], where orange/red depicts enrichment in the AFR group and blue depicts enrichment in the EUR group.

In contrast, there were substantially fewer DEGs when comparing AFR T versus EUR T (23 EUR-associated and 50 AFR-associated) or AFR N versus EUR N (18 EUR-associated and 56 AFR-associated). Only the pseudogene *H19* was consistently associated with AFR genetic similarity in both the AFR T versus EUR T and AFR N versus EUR N comparisons (Fig. 3A). Other DEGs were significant in either AFR T versus EUR T or AFR N versus EUR N, suggesting an interaction effect of genetic similarity with tumor/normal status. To further explore genetic similarity–associated differences, we compared AFR T and EUR T in the propensity-matched TCGA cohort (*n* = 147 EUR T and *n* = 49 AFR T). We identified 8,739 DEGs (3,787 upregulated in EUR and 4,952 upregulated in AFR), with the larger number of DEGs potentially reflecting differences in sample size and differential expression analysis formula design (note that comparison with normal tissue was not possible in the TCGA cohort, as only two patients with AFR genetic similarity had normal tissue available). Of the DEGs identified in the JHU AFR T versus EUR T comparison, 34 were also significantly associated with AFR and EUR groups in the TCGA analysis (labeled in Fig. 3A and B). Several of these genes have been associated with immune modulation in the context of cancer, including *Protein Arginine Deiminase 1* (*PADI1*) and the immune checkpoint *VCTN1* (encoding B7-H4; refs. 26, 27). Hallmark GSEA revealed differential enrichment of 18 out of 50 hallmark gene sets in the JHU analysis (2 enriched in AFR and 16 enriched in EUR) and differential enrichment of 32 out of 50 hallmark pathways (all enriched in EUR) in the TCGA analysis. Comparison of GSEA results between the JHU and TCGA cohorts revealed substantial overlap, with 13 of 18 gene sets showing consistent differential enrichment across both cohorts. In both cohorts, EUR ccRCC tumors were enriched in inflammatory pathways (IFN-α and IFN-γ response, allograft rejection), proliferative pathways (E2F targets, G_2_–M checkpoint, Myc signaling), metabolic pathways (glycolysis, oxidative phosphorylation, fatty acid metabolism, bile acid metabolism, peroxisome, and MTORC1 signaling), and the unfolded protein response. Conversely, no hallmark gene sets were consistently enriched in the AFR group.

**Figure 4:**
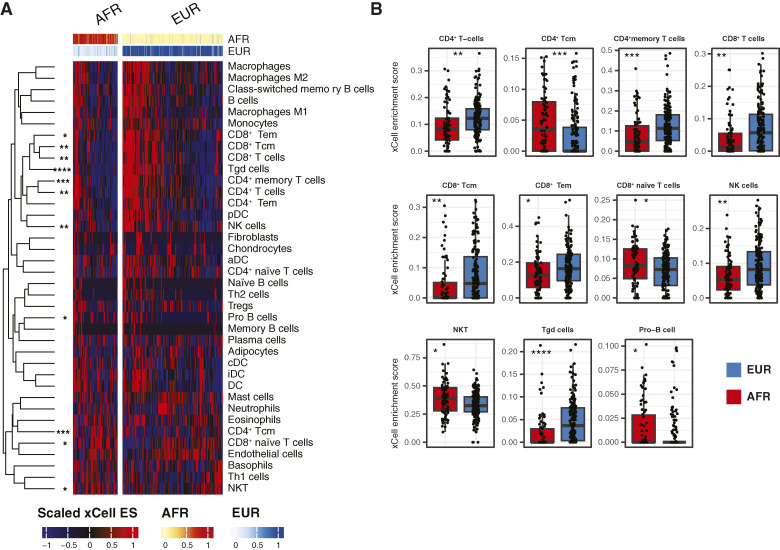
AFR and EUR groups are associated with a distinct tumor microenvironment composition. **A,** Heatmap of xCell results performed on the pooled analytic cohort (*n* = 78 AFR and *n* = 175 EUR). Values are row-scaled xCell enrichment scores for lymphoid, myeloid, and stromal cell types (*n* = 38 cell types). **B,** Box plots depict xCell enrichment scores for differentially enriched cell types. Box plot borders depict the median, upper quartile, and lower quartile. *P* values were determined by the Kruskal–Wallis test with multiple hypothesis correction. Statistical significance is as follows: *, *P* <0.05; **, *P* < 0.01; ***, *P* < 0.001; ****, *P* < 0.0001. aDC, activated dendritic cell; cDC, conventional dendritic cell; DC, dendritic cell; ES, enrichment score; iDC, immature dendritic cell; NKT, NK T cell; pDC, plasmacytoid dendritic cell; Tcm, central memory; Tem, effector memory; Tgd, gamma–delta T cell; Treg, regulatory T cell.

Given the consistent enrichment for inflammatory pathways across datasets, we performed cellular deconvolution using xCell (21), which produces enrichment scores for 64 cell types. Our analysis focused on lymphoid, myeloid, and stromal cell types (*n* = 38) and compared enrichment scores across AFR and EUR groups in the pooled analytic cohort (AFR = 78, EUR = 175; Fig. 4). After multiple hypothesis testing corrections, we found significant associations between the AFR/EUR group and 11 cell type signatures (FDR < 0.05). The EUR group was enriched for CD4^+^, CD8^+^ T cells, NK cells, and gamma–delta T cells, whereas the AFR group was enriched for naïve CD8^+^ T cells, CD4^+^ T central memory, NK T cells, and pro–B-cell signatures (FDR < 0.05 for all comparisons).

### Genetic similarity is associated with a distinct distribution of ccRCC molecular subtypes

We next sought to evaluate the impact of genetic similarity on gene expression in the context of the previously defined IMm151 ccRCC molecular subtypes: “Angiogenic,” “Angiostromal,” “Complement/Omega-Oxidative (Omega-Ox),” “T-Effector (T-Eff)/Proliferative,” “Proliferative,” and “Stromal/Proliferative.” These transcriptomically defined subtypes exhibited distinct somatic mutation and tumor microenvironment profiles (22). Furthermore, they demonstrated predictive potential in the metastatic setting, leading to their ongoing evaluation as biomarkers for treatment selection in the prospective setting (OPTIC RCC, NCT05361720; ref. 28). We asked if the differential distribution of the IMm151 subtypes across AFR and EUR groups could account for the transcriptomic and somatic variation observed above. To test this, we developed a random forest classifier to predict the IMm151 molecular subtype using deposited RNA-seq data from the original IMm151 study (*n* = 795 samples) and applied it to the pooled JHU and TCGA cohorts (*n* = 253 with RNA-seq, AFR = 78, EUR = 175). The classifier reached an overall accuracy of 91.1% and was most accurate in classifying “Angiogenic” (96.8%), “Angiogenic/Stromal” (94.4%), and “T-Eff/Proliferative” (95.4%) subtypes. Accuracy ranged between 81.5% and 92.4% for the remaining subgroups though specificity was >95% in all subtypes (Supplementary Fig. S4A and S4B). Inspection of subtype-defining genes supported the consistency of classification with the original study. For instance, high expression of angiogenesis genes (*VEGFA*, *KDR*, *ESM1*, *CD34*, *PECAM1*, and *ANGPTL4*) was shared by the “Angiogenic” and “Angiogenic/Stromal” groups, but they were distinguished by the expression of stromal genes (*FAP*, *FN1*, *COL5A1*, *COL5A2*, *POSTN*, *COL1A1*, *COL1A2*, *MMP2*) in the latter. The “T-Eff/Proliferative” group had the highest level of effector T-cell (*CD8A*, *IFNG*, *EOMES*, *PRF1*, *CD274*) and cell-cycle (*CDK2*, *CDK4*, *CDK6*, *BUB1*, *BUB1B*, *CCNE1*, *POLQ*, *AURKA*, *MKI67*, *CCNB2*) genes (Supplementary Fig. S4C).

**Figure 5:**
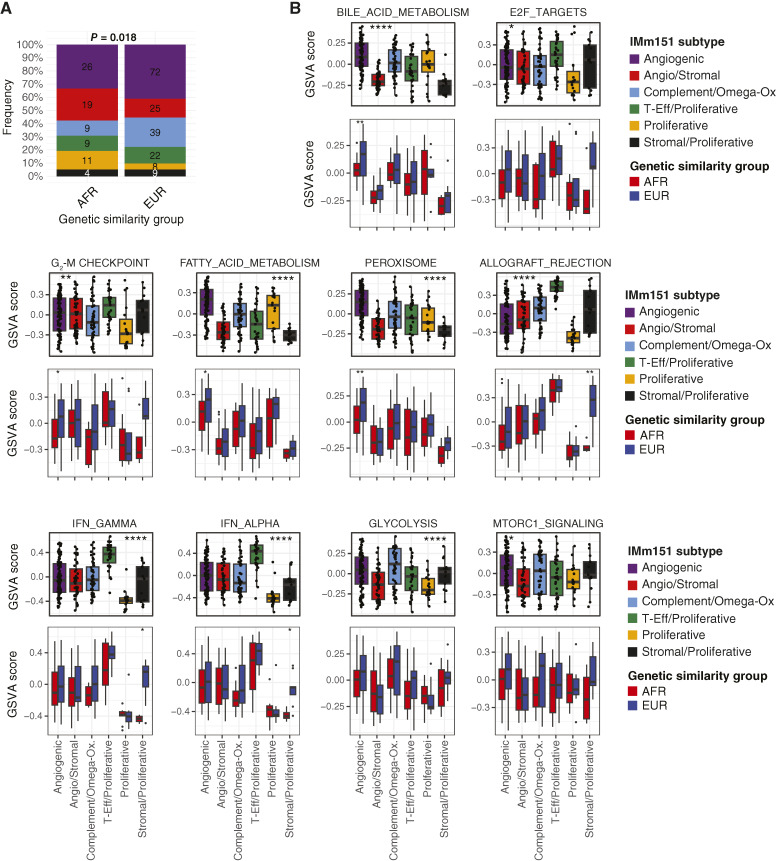
AFR and EUR groups are associated with distinct ccRCC molecular subtypes. **A,** Distribution of IMm151 molecular subtypes among AFR and EUR groups. **B,** GSVA of differentially enriched hallmark gene sets by IMm151 subtype (top) and further subsetted by genetic similarity (bottom). Box plots depict the median, upper quartile, and lower quartile GSVA scores. *P* values were calculated by the Kruskal–Wallis test. Statistical significance is as follows: *, *P* < 0.05; **, *P* < 0.01; ***, *P* < 0.001; ****, *P* < 0.0001.

We observed a significant difference in the distribution of IMm151 molecular subtypes between the AFR and EUR groups (*P* = 0.018). The AFR group contained a greater frequency of the “Proliferative” [AFR = 11 (14.1%) vs. EUR = 8 (4.6%)] and “Angio/Stromal” [AFR = 19 (24.4%) vs. EUR = 25 (15.9%)] subtypes, while the EUR group contained a greater frequency of the “Complement/Omega-Ox” [AFR = 9 (11.5%) vs. EUR = 39 (22.3%)] and “Angiogenic” [AFR = 26 (34.7%) vs. EUR = 72 (41.1%)] subtypes (Fig. 5A). Next, we assessed whether the differential membership across ccRCC IMm151 subtypes could explain the differentially enriched hallmark gene sets observed in Fig. 3C. We performed GSVA using the hallmark gene sets found to be differentially enriched between AFR T and EUR T specimens in both the JHU and TCGA cohorts. Using this approach, 10 of 13 gene sets were differentially enriched by the AFR/EUR group (Supplementary Fig. S5). These included the inflammatory pathways (IFN-γ, IFN-α, and allograft rejection), proliferative pathways (E2F and G_2_–M), and metabolomic pathways (bile acid metabolism, fatty acid metabolism, glycolysis, MTORC1 signaling, and peroxisome), all of which were enriched in EUR relative to AFR cohorts. As expected, the IMm151 subtypes were strongly associated with hallmark pathway GSVA scores. The “T-Eff/Proliferative” subtype was enriched for IFN-α, IFN-γ, and allograft rejection gene sets (*P* < 0.0001 for all). The “Proliferative” subtype, on the other hand, had the lowest enrichment for these gene sets. The “Angiogenic” and “Complement/Omega-Ox” subtypes were enriched in fatty acid metabolism, bile acid metabolism, peroxisome, and glycolysis gene sets (*P* < 0.0001; Fig. 5B). Given the relatively high fraction of the “Proliferative” subtype and low fraction of the “Angiogenic” and “Complement/Omega-Ox” subtypes in the AFR group, we suspected that IMm151 subtype classification may explain the enrichment of these hallmarks in the EUR group. Consistent with this, after controlling for IMm151 molecular subtype, few AFR/EUR group–related differences persisted. Notable exceptions included the enrichment of peroxisome (*P* < 0.01), G_2_–M checkpoint (*P* < 0.05), bile acid metabolism (*P* < 0.01), and fatty acid metabolism (*P* < 0.05) gene sets in the EUR group within the “Angiogenic” subtype and enrichment for IFN-γ (*P* < 0.05), IFN-α (*P* < 0.05), and allograft rejection (*P* < 0.01) gene sets among the EUR group within the “Stromal/Proliferative” subtype. Although genetic similarity–related differences in the “Stomal/Proliferative” subtype might be attributed to sample size (*n* = 4 AFR and *n* = 9 EUR), this is less likely to be the case among the “Angiogenic” subtype (*n* = 26 AFR and *n* = 72 EUR).

### AFR/EUR group and ccRCC molecular subtype are associated with *VHL* mutation frequency

**Figure 6:**
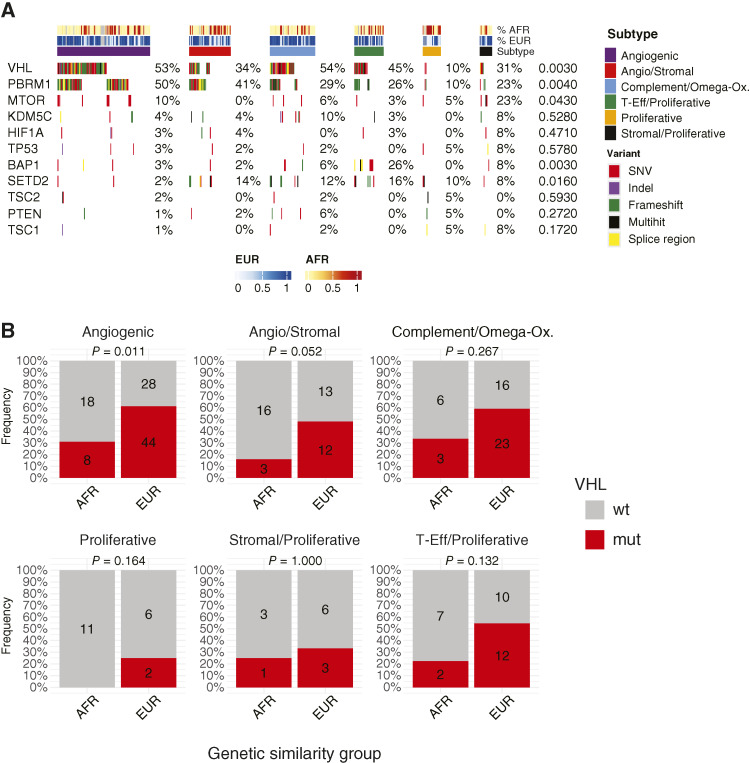
*VHL* mutation status is associated with both molecular subtype and genetic similarity. **A,** OncoPrint of ccRCC driver mutations split by IMm151 molecular subtype. *P* values were calculated using Fisher’s exact test. **B,** Frequency of *VHL* mutations in patients by IMm151 molecular subtype and AFR/EUR grouping. *P* values were calculated using Fisher’s exact test. Mut, mutant; SNV, single nucleotide variant; wt, wild-type.

We next performed an integrative analysis of somatic mutations with molecular subtype for 237 samples with both transcriptomic and somatic mutation data (Fig. 6). The distribution of somatic driver mutations across IMm151 subtypes was consistent with the original publication (22). For example, *VHL* (*P* = 0.003) and *PBRM1* (*P* = 0.004) mutations were highest among the “Angiogenic” subtype and lowest among the “Proliferative” subtype, whereas *BAP1* mutations were enriched in the “T-Eff/Proliferative” subtype (*P* = 0.003). We then assessed whether the differential frequency of *VHL* mutations by AFR/EUR group was explained by the differing distribution of ccRCC molecular subtypes (Fig. 6B). We found that among all subgroups, the frequency of *VHL* mutations was numerically lower among AFR versus EUR groups, and this reached statistical significance in the “Angiogenic” (*P* = 0.011) subtype.

## Discussion

We explored the associations of AFR and EUR genetic similarity with the molecular landscape of ccRCC by performing an integrative molecular analysis of a case–case-matched cohort of B and W individuals. Although several studies have explored race- and ancestry-associated differences among ccRCC cases in TCGA (7, 8, 10), we build upon these studies by generating an independent cohort and controlling for potential confounders, including histologic subtype, disease stage, grade, and gender (29–31). Through the use of our own data and publicly deposited data, we report findings on the largest cohort of ccRCC patients with AFR genetic similarity to date. We confirm a significant association of *VHL* mutation and chromosome 3p deletion frequency with EUR genetic similarity, and we also identify distinct gene expression patterns and tumor microenvironment compositions associated with AFR and EUR groups. We contextualize these findings through the application of recently defined ccRCC molecular subsets.

From a somatic alteration standpoint, we found a decreased frequency of *VHL* mutations and 3p loss in patients with predominantly AFR genetic similarity relative to EUR genetic similarity, even when correcting for other potentially confounding clinical variables such as HTN and eGFR. These observations suggest that race and AFR/EUR group, rather than these comorbidities—which are more frequent in B individuals—contribute to the differences in somatic alterations (25). Importantly, in a pooled cohort, after correcting for multiple hypothesis testing, deletions in chromosome 3p and *VHL* mutations were the only statistically significant somatic alterations that differed between the AFR and EUR groups. We did not identify any alterations that were enriched in the AFR cohort, potentially reflecting our limited sample size. As AFR and EUR genetic similarity and race are highly correlated, we next examined AFR percentage and its association with *VHL* mutations among patients with B race and found no statistically significant association. This suggests that the observed differences are more closely linked to the AFR and EUR groups and race rather than to gradations of AFR genetic similarity within individuals. This would be consistent with a noninheritable etiology of these differences in the somatic mutation landscape though limited sample size could influence these results.

Our transcriptomic analyses revealed that different genes are altered by the AFR/EUR group when comparing normal renal tissue versus ccRCC tumors, indicating an interaction between genetic similarity and tissue status. This is consistent with findings from the Genotype-Tissue Expression project, which identified a substantial number of genes differentially associated with clinical variables such as ancestry group, sex, and body mass index across healthy tissues (32). We focused our analysis on DEGs and differentially enriched gene sets associated with the AFR and EUR groups specifically in tumor samples. When comparing genetic similarity–related differences in ccRCC specimens in both JHU and TCGA datasets, we found that the EUR group was consistently enriched for pathways associated with inflammation (e.g., IFN-α and IFN-γ responses, allograft rejection), proliferation (e.g., E2F targets, G_2_–M checkpoint), and metabolism (e.g., glycolysis, bile acid metabolism, fatty acid metabolism, peroxisome, MTORC1 signaling). Notably, previously reported differences in hypoxia signaling were only evident in the TCGA cohort (11). Supporting differences in inflammatory signaling, cellular deconvolution revealed a distinct inflammatory composition across EUR T and AFR T. Consistent with this, EUR genetic similarity was associated with a higher estimated proportion of CD8^+^ T cells and NK cells. Among the top genes associated with the AFR/EUR group, *PADI1* and *VCTN1* (encoding B7-H4) were both associated with AFR genetic similarity in both the JHU and TCGA cohorts. The proteins encoded by these genes have both been shown to modulate the TME (26, 27) and may contribute to the different TME compositions observed at the AFR/EUR group level. For example, the AFR group was enriched for naïve CD8^+^ T cells, CD4^+^ T central memory cells, NK T cells, and pro–B cells. Given the clinical use of immune checkpoint inhibitors in the clinical management of ccRCC, these differences may have clinical implications (33) though further studies are required to investigate the mechanisms and clinical significance.

We next asked if differential membership across ccRCC IMm151 molecular subtypes (22) could account for the genetic similarity–associated differences observed above. These subtypes have both biological and clinical relevance. From a clinical standpoint, they are currently being evaluated as potential biomarkers in a prospective trial (OPTIC RCC, NCT05361720; ref. 28). Furthermore, they have been shown to be associated with different metastatic disease distributions, suggesting a potential association with ccRCC organotropism (34). We found that the distribution of IMm151 subtypes was statistically significantly associated with the AFR/EUR group, with a higher prevalence of the “Proliferative” and “Angio/Stromal” subtypes in the AFR group and a higher prevalence of the “Complement/Omega-Ox” and “Angiogenic” subtypes in the EUR group. Upon reexamining the genetic similarity–based differences in the context of IMm151 subtypes, we found that most—but not all—variation in hallmark gene set pathway enrichment was explained by IMm151 subtype designation. Similarly, although the IMm151 subtype was strongly associated with *VHL* mutational status—with the “Proliferative” subtype demonstrating a reduced frequency of *VHL* mutations—we found that AFR genetic similarity was associated with a decreased frequency of *VHL* mutations across IMm151 subtypes, reaching statistical significance within the “Angiogenic” subtype. Given the expected transcriptional homogeneity within an IMm151 subgroup, this may indicate an alternative method of loss or inactivation (i.e., epigenetic, deletion, posttranslational), but further analyses are required to elucidate this. Conversely, in the “Proliferative” subtype, which had a very low *VHL* mutation rate, it is possible that these reflect true *VHL* wild-type disease. Distinguishing this is of clinical relevance given the clinical approval of belzutifan, a HIF-2α inhibitor, which presumably is only effective in *VHL*-mutant RCC (35).

It is worth noting that the IMm151 subtypes were discovered using nonnegative matrix factorization, an unsupervised machine learning model, performed on a cohort of 823 samples from a phase III clinical trial. Notably, there were very few (4 out of 823) patients with self-reported B race, and genetic similarity was unreported. The low representation of B individuals in this cohort may contribute to the additional variation in somatic mutation frequency and gene set enrichment explained by the AFR/EUR group and highlights the importance of including a representative cohort in training datasets for unsupervised models. Despite these limitations, the predicted IMm151 subtype was strongly associated with both transcriptomic and somatic variation, supporting the use of these classifiers over clinical variables to stratify patient outcomes. Given the low number of metastatic cases in our cohort, our ability to extrapolate the observed differences in molecular subtype membership by AFR or EUR genetic similarity to the metastatic space and thus assess the impact on systemic therapy responsiveness is limited, but it is an important future direction. Extending our analysis to a metastatic ccRCC cohort is particularly relevant given the proposed predictive significance of the IMm151 subtypes. Patients within the “Proliferative” subtype demonstrated poorer responses to VEGF inhibitors compared with other subgroups. Consistent with our observation that the “Proliferative” subtype is enriched among patients with AFR genetic similarity, a retrospective study comparing outcomes with VEGF inhibitors in metastatic ccRCC found inferior outcomes in self-reported B patients though this was not statistically significant when controlling for International Metastatic RCC Database Consortium score (36).

This study has notable limitations. First, the data generated in this study comprises a single institutional dataset and is relatively limited in size. This may introduce unknown biases that must be considered when interpreting results. We attempted to control for this by employing a case–case-matched approach and accounting for confounding clinical variables and recently defined molecular subtypes. Pooling genetic data from different sources (in-house sequencing and the TCGA) also presents technical challenges. Nonetheless, pooling samples from multiple datasets is necessary to augment the power of our study in examining underrepresented minorities. Second, we found that in the context of our study population, self-reported B and W race were strongly correlated with AFR and EUR 1000 Genomes groups, and thus, conclusions about associations with AFR or EUR are confounded by race. We attempted to mitigate this by examining the AFR percentage within self-reported B individuals and by assessing the association of potential confounding clinical variables (such as HTN or chronic kidney disease) with mutation status.

Despite these limitations, we establish a correlation between genetic similarity and the tumor somatic, immune landscape, and IMm151 molecular subtype of ccRCC. We suggest that differential membership across molecular subtypes provides a possible explanation for the observed molecular differences by race and/or genetic similarity in prior studies of ccRCC. Our study underscores the need to increase racial and ethnic diversity in molecular and clinical studies and to implement molecular subtyping clinically to facilitate more personalized approaches to management. To facilitate further research in this area, we make our data available to increase the number of AFR genetic similarity patients with ccRCC included in molecular analyses. Recognizing and addressing the molecular differences associated with AFR and EUR genetic similarity is essential for advancing precision oncology and improving outcomes for all patient populations.

## Supplementary Material

Supplementary Tables Supplementary Tables

Supplementary Figures Supplementary Figures
