# Supplementary material for: Clear-Cell Renal Cell Carcinoma Molecular Subtypes Differ by African and European Genetic Similarity
Source: Cancer Res Commun. 2025 May 1;5(5):743–55. doi: 10.1158/2767-9764.CRC-24-0624 (PMC12044083; doi:10.1158/2767-9764.CRC-24-0624)
Supplement: Supplementary Figures [file crc-24-0624_supplementary_figures_1-5_suppsf1-sf5.pdf]

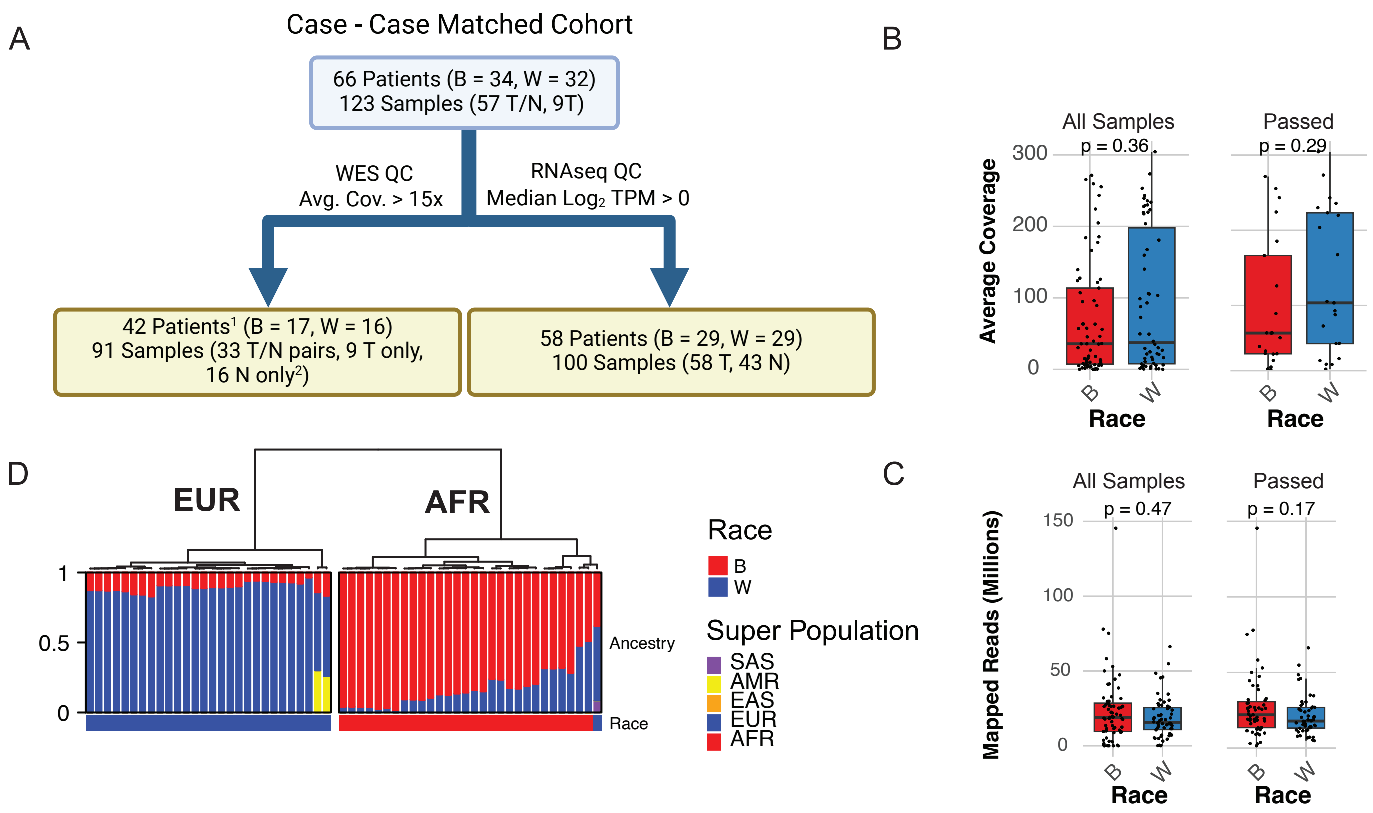

Supplementary Figure 1. JHU cohort overview and genetic similarity estimation. We submitted 123 samples from 66 patients for WES and RNAseq. (A) WES samples with an average coverage less than 15x were excluded from downstream analysis (n = 32), resulting in 91 samples (33 T/N pairs, 9 T only, 16 N only) which passed. [1]Patients with a passed tumor specimen enabling somatic mutation calling.[2]Normal specimens were used for downstream ancestry estimation. RNAseq samples with a median Log2 TPM= 0 were excluded, resulting in 102 samples (58 T, 44N) from 58 patients which passed. Created in BioRender.com (B) Box plot of WES average coverage and (C) RNAseq mapped reads of all and passed samples. There was no statistically significant difference in average coverage by race. Box plot borders depict the median, upper quartile, and lower quartile. P-values were calculated by the Kruskal-Wallis test. (D) Unsupervised hierarchical clustering of Euclidean distances calculated from ADMIXTURE genetic similarity estimates. The stacked bar plot indicates the percentage contribution of each continental population (as defined in 1000 Genome Project). Splitting at the top 2 cluster levels resulted in EUR and AFR cohorts. Abbreviations: AFR, African; AMR American; Avg. Cov., Average Coverage; B, Black; EAS, East Asian; N, Normal; QC, quality control; SAS South Asian; T, Tumor; TPM, transcripts per million; W, White; WES, Whole-exome sequencing.

A

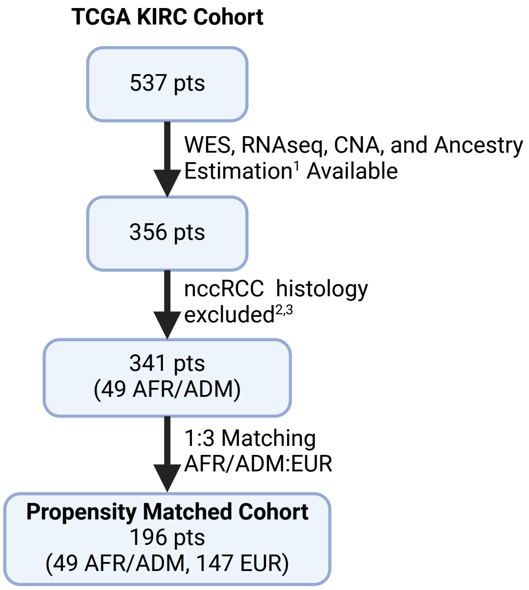

B

	EUR No. 147	AFR No. 49	P-value
Age	59.4 (±12.8)	59.3 (±10.7)	0.99
**Sex**			**0.51**
FEMALE	61 (41.5%)	23 (46.9%)	
MALE	86 (58.5%)	26 (53.1%)	
**Nuclear Grade**			**0.84**
G1	9 (6.1%)	4 (8.2%)	
G2	68 (46.3%)	20 (40.8%)	
G3	61 (41.5%)	21 (42.9%)	
G4	6 (4.1%)	2 (4.1%)	
GX	3 (2.0%)	2 (4.1%)	
**Stage**			**0.91**
NA	1 (0.7%)	1 (2.0%)	
Stage I	103 (70.1%)	34 (69.4%)	
Stage II	11 (7.5%)	4 (8.2%)	
Stage III	13 (8.8%)	4 (8.2%)	
Stage IV	19 (12.9%)	6 (12.2%)	
**Race**			**< 0.0001**
B	0 (0.0%)	48 (98.0%)	
W	144 (98.0%)	1 (2.0%)	
NA	3 (2.0%)	0 (0.0%)	

C

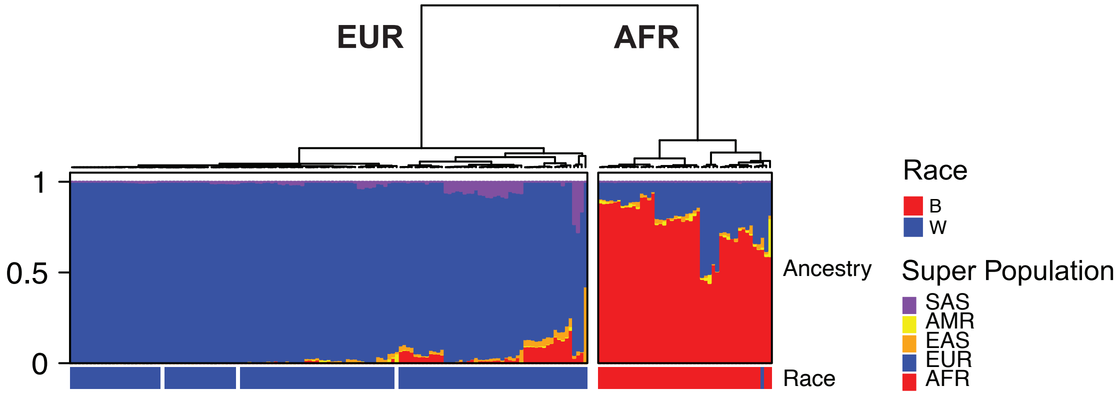

Supplementary Figure 2. Propensity matched TCGA KIRC cohort. (A). Consortium diagram of cohort inclusion/exclusion criteria. There are 537 patients within the TCGA-KIRC cohort accessible through the genomic data commons (gdc.cancer.gov). Of these, 356 had WES, RNAseq, Copy Number Analysis, and estimated ancestry. Non clear cell RCC histologies have been identified in prior publications utilizing the TCGA, we excluded these samples leaving 341 patients. This resulted in 49 patients with AFR or admixed ancestry, and we performed 1:3 matching to generate a final cohort of 196 patients. Created in Biorender.com (B) Baseline clinical characteristics of the propensity matched cohort. (C) Unsupervised hierarchical clustering of euclidean distances calculated of ADMIXTURE ancestry estimates. The stacked bar plot indicates the percentage contribution of each super population (as defined in 1000 Genome Project). Splitting at the top 2 cluster reveals an AFR and EUR enriched cohort. Abbreviations: AFR, African; AMR American; CNA, Copy Number Analysis; EAS, East Asian; SAS South Asian; WES, Whole-exome sequencing. References: 1) Carrot-Zhang et al., Cancer Cell, 2020. 2) Ricketts et al., Cell Reports, 2018. 3) Bakouny et al., Cell Reports, 2022.

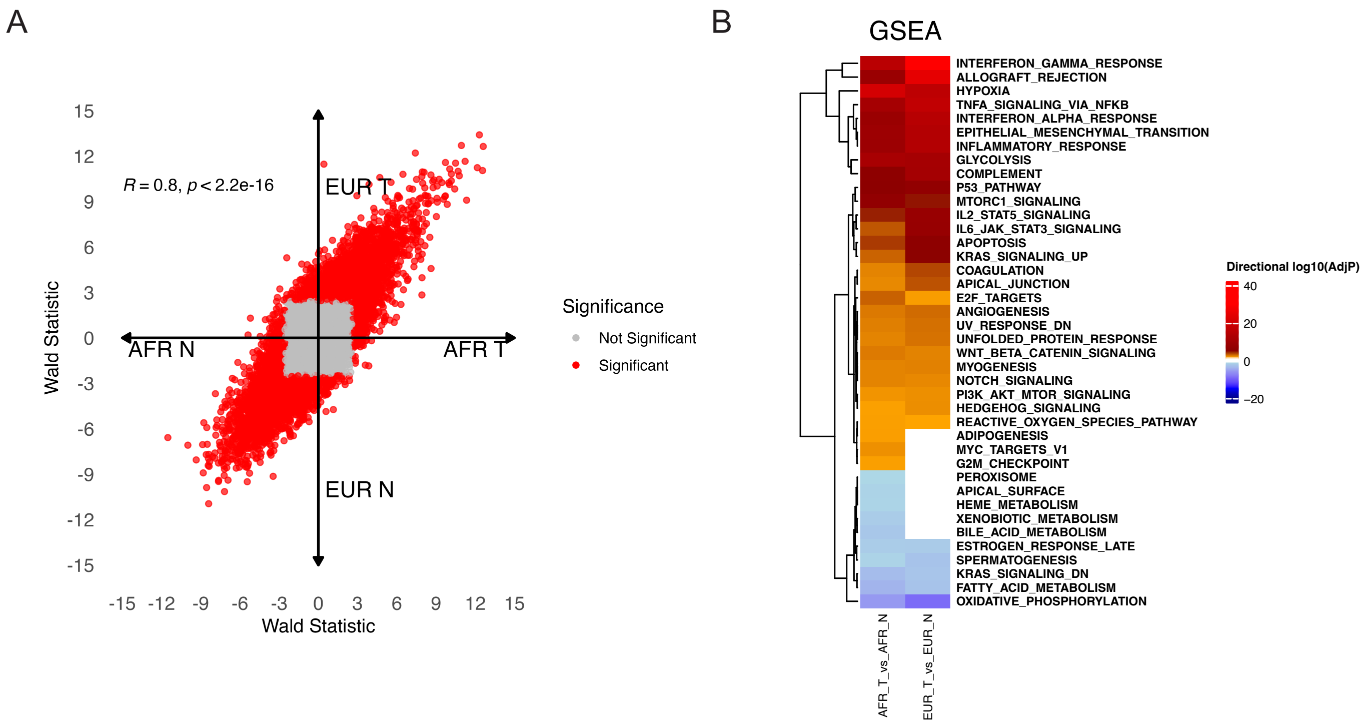

Supplementary Figure 3. Differential expression analysis of tumor vs normal in AFR and EUR groups. (A) Wald statistic from differential expression analysis of AFR tumor (T) vs normal (N) (x-axis) and EUR T vs N (y-axis), demonstrating strong correlation in the two conditions. Each point represents a gene, genes in red have a multiple hypothesis corrected p value < 0.05 in at least one of the comparisons. Correlation (R) and p value calculated by Pearson. (B) Heatmap of hallmark gene set enrichment results performed on the DEG lists from AFR T vs N and EUR T vs N. Plotted values are directional –Log10(Adj P), where orange/red depicts enrichment in tumors and blue depicts enrichment in normal tissue.

A

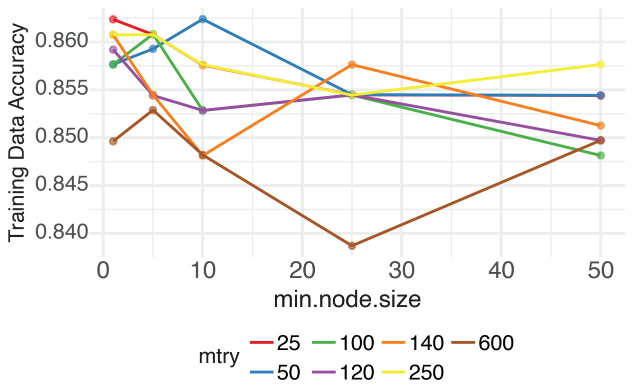

B

Test Dataset Model Performance

**Molecular Cluster**	Sensitivity	Specificity	Balanced Accuracy
Angiogenic	1	0.946	0.973
Angiogenic/Stromal	0.889	1	0.944
Complement/Ω-Oxidation	0.879	0.969	0.924
Proliferative	0.636	0.993	0.815
Stromal/Proliferative	0.708	1	0.854
T-effector/Proliferative	0.96	0.949	0.954
**Overall Performance**			0.911

C

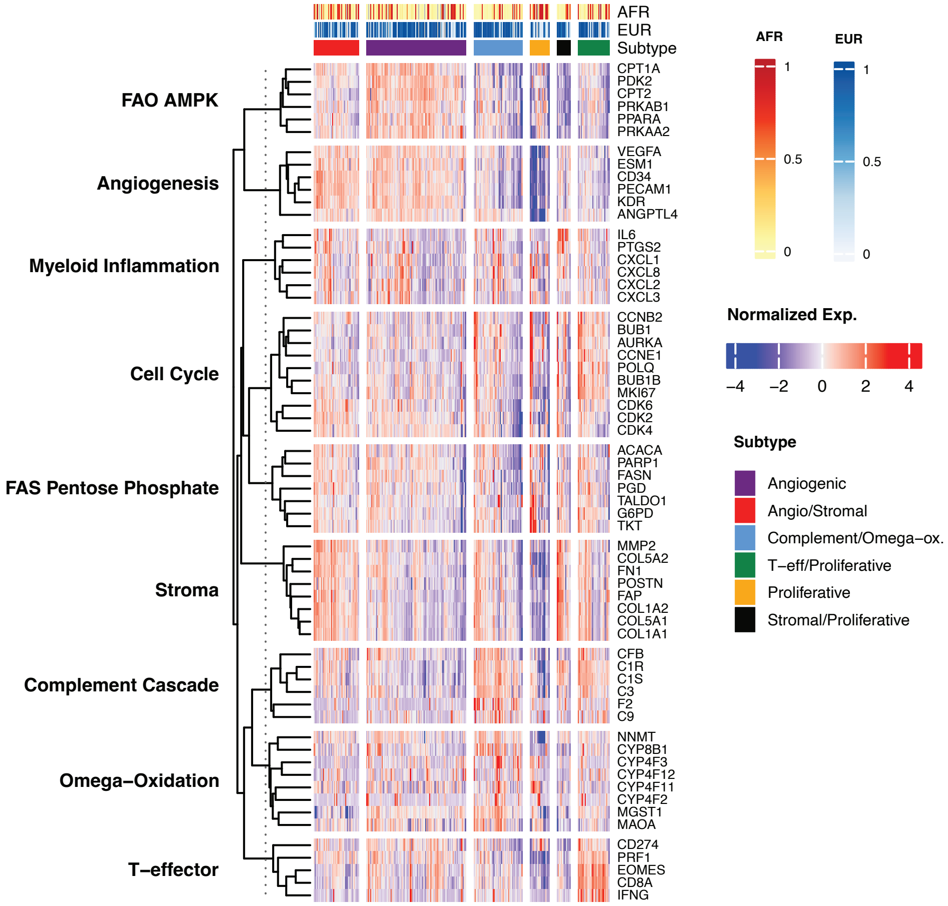

Supplementary Figure 4. (A) Random forest tuning assessing the parameters 'mtry', 'min.node.size'. Model was tuned in training cohort (n = 633) (B) Model performance in validation cohort (n = 162). (C). Heatmap of IMm151 ccRCC molecular subtype defining genes. Pooled log2 transformed transcripts per million are row normalized.

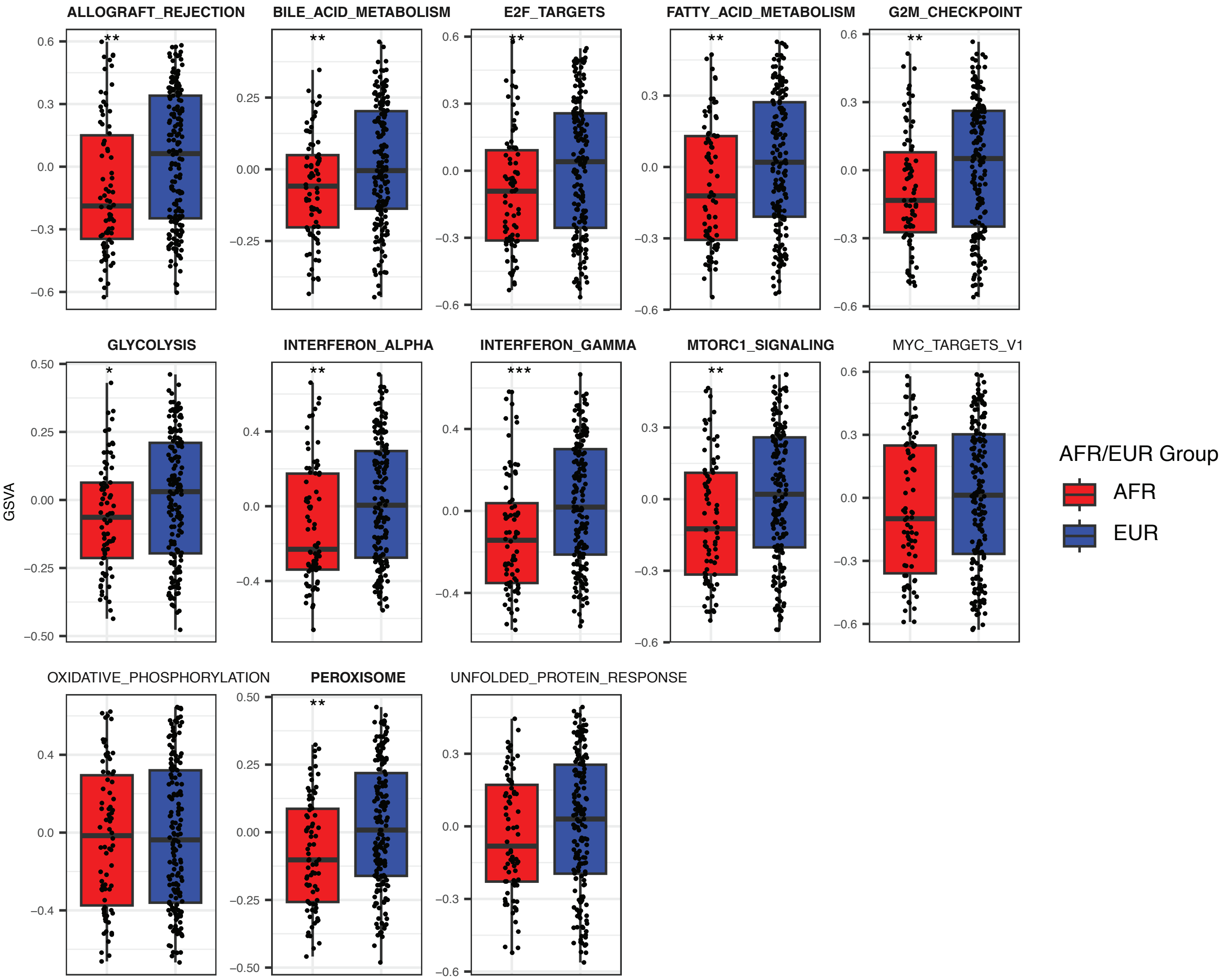

Supplementary Figure 5. Gene set variation analysis (GSVA) of the pooled cohort performed on differentially enriched hallmark gene sets across genetic similarity group (from Figure 3). Box plots depict the median, upper quartile, and lower quartile GSVA scores. P-values were calculated by the Kruskal-Wallis test. Statistical significance as follows: '*', p < 0.05; '**', p < 0.01; '***' < 0.001; '****' < 0.0001.
